# Advances and Prospects in the Study of Spherical Polyelectrolyte Brushes as a Dopant for Conducting Polymers

**DOI:** 10.3390/molecules29061315

**Published:** 2024-03-15

**Authors:** Na Su

**Affiliations:** Department of Printing and Packaging Engineering, Shanghai Publishing and Printing College, Shanghai 200093, China; suna@whu.edu.cn

**Keywords:** spherical polyelectrolyte brushes, conducting polymers, dopants, doping mechanism

## Abstract

Owing to their special structure and excellent physical and chemical properties, conducting polymers have attracted increasing attention in materials science. In recent years, tremendous efforts have been devoted to improving the comprehensive performance of conducting polymers by using the technique of “doping.” Spherical polyelectrolyte brushes (SPBs) bearing polyelectrolyte chains grafted densely to the surface of core particles have the potential to be novel dopant of conducting polymers not only because of their spherical structure, high grafting density and high charge density, but also due to the possibility of their being applied in printed electronics. This review first presents a summary of the general dopants of conducting polymers. Meanwhile, conducting polymers doped with spherical polyelectrolyte brushes (SPBs) is highlighted, including the preparation, characterization, performance and doping mechanism. It is demonstrated that comprehensive performance of conducting polymers has improved with the addition of SPBs, which act as template and dopant in the synthesis of composites. Furthermore, the applications and future developments of conductive composites are also briefly reviewed and proposed, which would draw more attention to this field.

## 1. Introduction

**Figure 1:**
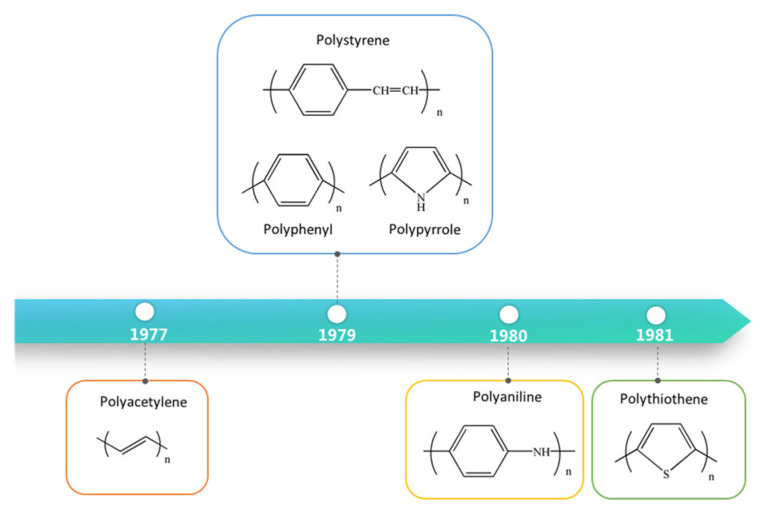
Typical conducting polymers.

Since p-doped polyacetylene with an electrical conductivity comparable to that of metal was first discovered in 1977 by Heeger, MacDiarmid and Shirakawa [1], organic polymers have not been considered as insulators. Accordingly, a series of conducting polymers such as polyaniline (PANI) [2,3], polypyrrole (PPy) [4,5], polythiophene (PT) [6,7,8] and their derivatives have emerged. Typical conducting polymers are illustrated in Figure 1.

As a π-conjugated polymer, the electrical conductivity of conducting polymers can be extended from insulator to conductor by chemical [9,10] or electrochemical doping [11,12]. In addition to polymer structure, the structure of conducting polymers also contains monovalent anion (p-type doping) or cation (n-type doping) introduced by doping. Therefore, conducting polymers not only have the characteristics of polymer itself but also the characteristics of metal (high conductivity) and semiconductor (p- and n-type) brought by doping. Stable aromatic ring structure has almost no conductivity. Although the quinone structure has high energy, it is less likely to exist. After doping, polaron and bipolaron appear gradually. With increased bipolarized substructures, a close to quinone structure forms, indicating the character of conductivity. The main characteristics of conducting polymer are as follows:

① The room temperature electrical conductivity of conducting polymers can change in the states of metal-insulator-semiconductor, which is unmatched by any other materials to date. ② The process of the doping and de-doping of conducting polymers is completely controllable. Using this unique property, conducting polymers can be used as gas or biosensor with high selectivity, high sensitivity and good repeatability. ③ The doping of conducting polymers is essentially controllable redox reaction. Therefore, the electrochromic or photochromic properties of conducting polymers may be engineered widely. It can be used not only in information storage and display, but also in camouflage and stealth of military targets. ④ Due to their π-conjugate structure, conducting polymers have a fast response time. 

In summary, as a new type of functional material with excellent physical and chemical properties, conducting polymers have far-reaching application potential in industrial production, such as metal corrosion-resistant materials [13,14], microwave absorbing materials [15,16], electrical materials [17,18,19], sensitive materials [20,21], fuel cells and water electrolyzers [22,23,24]. Researchers are committed to the practical application of conducting polymers, which means improving comprehensive properties and reducing costs by doping. Accordingly, the selection of novel and effective dopants is essential for conducting polymers.

**Figure 2:**
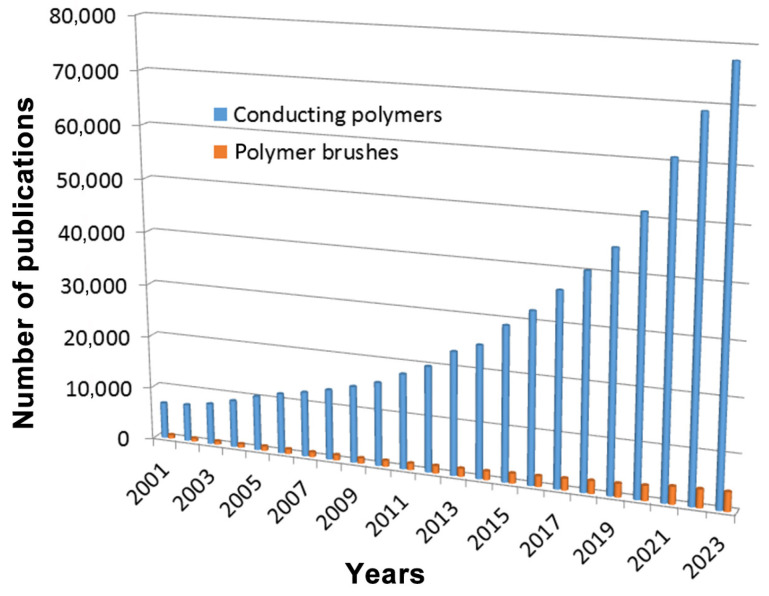
Number of publications in ScienceDirect database with Conducting polymers and Polymer brushes as keywords.

Spherical polyelectrolyte brushes (SPBs) that have cores particles and brush polymers with certain functional groups are promising novel and efficient dopants for conducting polymers. The growth direction for conducting polymers is provided by its spherical structure, and the properties of conducting composites are well controlled through the choice of grafted polymer chains and cores particles. The number of publications in the ScienceDirect database with conducting polymers and spherical polyelectrolyte brushes is shown in Figure 2.

To date, a series of reviews about conducting polymers have focused on synthesis [25], and their applications in electrochemical capacitive energy storage [26], Li-ion batteries [27], electrochemistry [28], biomedical [29] and electrochemical sensors and biosensors [30]. As for SPB, since the first review reported by Ballauff, M. in 2007 [31], many published reviews have mainly concentrated on theory and modeling [32], ionic effects [33] and their emerging application [34] in wet-end papermaking [35] and nanoreactors [36]. However, there is no review related to SPBs as the dopant of conducting polymers, which is still needed. In this review, a recent advancement on various dopants of conducting polymer is firstly introduced. Then, a survey of recent research on SPBs is presented, including the synthesis and characterization methods and conformation. In the following section, taking polypyrrole and polyaniline as examples, various literatures have reported that polyaniline, polypyrrole and poly(pyrrole-co-aniline) doped with SPBs have been prepared and characterized, which reveals that the performance of conductive composites is closely related to the structure of SPBs. In addition, the doping and conducting mechanisms of SPBs are discussed in detail, which provides theoretical support for SPBs as novel dopant for conducting polymers. Furthermore, the applications of conducting polymers doped by SPBs are summarized, and an outlook towards future development is provided.

## 2. Dopants of Conducting Polymers

### 2.1. Acids

Currently, acid doping is the most common method used in the synthesis of conducting polymers. Inorganic acid doping often only has a significant effect in improving the electrical conductivity of conducting polymers. To obtain conducting polymers with controllable microstructures, a template or a soft-template is added while doping with inorganic acid. Common dopants are hydrochloric acid, aromatic sulfonic acid derivatives (such as dodecyl benzene sulfonic acid (DBSA) [37,38]) and p-toluenesulfonic acid (TsOH) [39,40].

Lee J et al. [41] developed a surface-active DBSA as an anionic additive in 1995. Solubilization of polypyrrole was achieved in the aqueous solution of ammonium persulfate (APS) by oxidative polymerization of pyrrole monomers. Subsequent studies have further suggested that the addition of other sulfonic acid compounds which possess surface-active characteristics can also cause the solvation of polypyrrole [42]. Omastovà M et al. [43] synthesized conductive polypropylene/clay/polypyrrole (PP/clay/PPy) nanocomposites by chemical oxidative polymerization using dodecylbenzenesulfonic acid (DBSA) as dopant and ferric chloride (FeCl_3_) as oxidant. Yin W S and Ruckenstein E [44] explored HCl and DBSA co-doped soluble polyaniline, whose electrical conductivity was up to 13.9 S/cm. Shen Y Q and Wan M X [45] synthesized soluble polypyrrole using DBSA-CSA mixed acid as dopant, and its room temperature electrical conductivity ranged from 2 S/cm to 18 S/cm. The electrical conductivity of PPy increased with the increase of CSA contents, and DBSA and CSA contributed to the dissolution of PPy during mixed acid doping. They believed that the reason might be that DBSA reduced the interaction between PPy molecular chains, while long alkyl chain of DBSA played a positive role in the dissolution of PPy.

### 2.2. Surfactants

When surfactants are added, the rich morphology of conducting polymers can be attributed to their special molecular structure. Surfactants such as organic sulfonates (e.g., SDS, SDBS) [46,47], polyvinylpyrrolidone (PVP) [48,49] and sodium p-toluenesulfonate (TsONa) [50,51] are commonly used.

Yusuke H et al. [52] illustrated the preparation of PPy nanopowders using anionic surfactants (SDS, SDBS, CTAB) by emulsion polymerization. The surfactants affected the electrical conductivity of the nanopowders. The electrical conductivity was at 10 S/cm when SDS and SDBS were added. The electrical conductivity was increased with increasing SDBS content. Monodisperse PPy nanoparticles with the particle sizes of 35~102 nm were produced at a stirring rate of 13,500~24,000 rpm. The electrical conductivity of PPy using CTAC as surfactant was lower than that of SDS and SDBS. In addition, Zhou D H et al. [53] demonstrated the fabrication of PANI nanofibers with high electrical conductivity in the presence of mixed surfactants CTAB-SDBS. These mixed surfactants acted as soft-template and a dispersant. The effect of CTAB-SDBS ratio on the morphology and electrical conductivity of PANI was investigated. When the molar ratio of CTAB to SDBS was 2:1, the electrical conductivity reached 0.102 S/cm, which was two orders of magnitude higher in the case of surfactant alone. Feng X M et al. [54] synthesized AgCl/PANI core-shell nanocomposites using the one-step method in the presence of PVP. The composites with a core diameter of approximately 20–50 nm and shell thickness of 20 nm were obtained in the percentage concentration of 4% PVP. The nanocomposites had a uniform particle size and good dispersibility.

### 2.3. Inorganic Nanoparticles

Based on the theory of mechanical reinforcement of polymer composites, their mechanical properties can be enhanced by adding inorganic additives. In order to improve the thermal stability and processability of conducting polymers, inorganic nanoparticles (e.g., Fe_3_O_4_, Fe_2_O_3_, SiO_2_ and NiO) are utilized. Inorganic nanocomposites such as PPy/Fe_3_O_4_ [55], PPy/SiO_2_ [56], PANI/NiO [57] and PANI/ Fe_2_O_3_ [58] are reported.

Chen W et al. [59] developed the Fe_3_O_4_-PPy nanocomposites with magnetic and electrical conductivity by applying a PPy conductive layer on the surface of magnetic nanoparticles (Fe_3_O_4_). The average particle size of Fe_3_O_4_-PPy nanocomposites was about 50 nm. With the increase of Fe_3_O_4_ content, the magnetic resistance increased from 98.4 Oe to 116.3 Oe, the saturation magnetization increased from 0.268 to 9.23 emu/g and the electrical conductivity increased from 10^−5^ to 10^−2^ S/cm. Liu X H et al. [60] synthesized SiO_2_/PPy core-shell particles with controllable shell thickness. The shell thickness increased as the amount of pyrrole monomer increased. The composites with different properties and functions were synthesized using monodisperse SiO_2_ microspheres as templates. Shambharkar B H and Umare S S [61] successfully prepared PANI/NiO nanocomposites. The electrical conductivity, magnetic properties and thermal stability of PANI were influenced by the addition of NiO. The electrical and magnetic properties of the composites were determined by the size and concentration of NiO added. The thermal stability of the composites was improved by the addition of NiO. These occurrences were possibly due to the fact that the grain boundary effect between the NiO particles and the PANI backbone restricted thermal motion of the PANI in the composite. Along with that, due to this interaction, lattice distortion occurs around the doped NiO particles. Accordingly, the charge trappings become stronger, thus facilitating efficient electron transport.

Furthermore, taking carbon materials involving carbon nanotubes [52,53,54,55,56,57,58,59,60,61,62,63,64], carbon nanofibers [65] and graphene [66,67] as dopant may improve the performance of conductive polymers because of the excellent electrical, mechanical and chemical properties. Jeon I Y et al. [68] studied PPy-g-MWCNT nanocomposites by grafting multi-walled carbon nanotubes (MWCNT) onto polypyrrole. The electrical conductivity of PPy-g-MWCNT nanocomposites was 20 times higher than that of PPy after alkali treatment, and its current density and cycle stability increased, which indicated that the electrons were effectively transmitted through covalent bonds.

### 2.4. Polymers

Recently, polyelectrolytes have been successfully used as the dopants of conducting polymers to fabricate conducting composites [69,70,71]. Goel S [72] developed a simple template-free interface polymerization method to synthesize PPy nanofibers using HCl, FeCl_3_, p-toluenesulfonic acid (p-TSA), camphor sulfonic acid (CSA) and polystyrene sulfonic acid (PSSA) as dopants, respectively. Results showed that the contributions of each dopant on thermal stability and electrical conductivity of PPy nanofibers followed the order PPy-p-TSA > CSA > HCl > FeCl_3_ > PSSA, PPy-p-TSA > CSA > HCl > FeCl_3_ > PSSA, respectively. When doped with PPy-p-TSA, the electrical conductivity of PPy nanofibers reached 6 × 10^−2^ S/cm. Wei J et al. [73] prepared PANI/POSS-PSS nanoparticles with core–shell structure via in-situ polymerization using star-like POSS-PSS as a dopant. The electrochromic device with PANI/POSS-PSS as the active layer exhibited better electrochromic performance than the device with linear PSS-doped PANI as the active layer. Mpoukouvalas K et al. [74] conducted in-depth research on polypyrrole-polystyrene sulfonate PPy/PSS_x_ (x = H^+^, Li^+^, Na^+^, Cs^+^) core-shell nanoparticles. Results showed that different cations were found to have different effects on the chemical behavior of PPy/PSSx system under the condition of the same volume fraction of PPy. Significantly different chemical behavior of H^+^, Na^+^ and Li+, Cs^+^ occurred at temperatures above 400 K, which might be due to the influence of ionic conductivity. For PPy/PSSx particles, the DC electrical conductivity at constant temperature decreased with the increase of the counter-ion radius (except for Li).

In addition, dye [75,76], cyclodextrin and its derivatives [77,78] have also been used as dopants in the polymerization of conducting polymers.

In summary, significant progress has been made in improving certain properties of conducting polymers, such as electrical conductivity, thermal stability, mechanical properties, solubility or processability. However, the aforementioned dopants display obvious disadvantages. Small-molecule dopants such as acids or surfactants are prone to de-doping, which leads to possible instability for the electrical conductivity of conductive composite. As for conducting polymers doped by inorganic nanoparticles and polymers, the improvement of thermal stability and processability may be at the cost of losing electrical conductivity. In addition, preventing agglomeration during the polymerization process is also a difficult task. 

## 3. Spherical Polyelectrolyte Brushes

Polymer brush refers to a special homopolymer or copolymer system formed by high-density coupling of one end of polymer chains to various interfaces [79]. Basic understanding of polymer brush can be traced back to the early theoretical studies by Pincus [80] and Borisov [81]. Subsequently, numerous theoretical models [82,83,84] have been explored. 

For the prospect of theory, the configuration of polymer chains depends on the interaction between polymer brushes. The balance between the interaction among polymer chains and their elastic free energy is reflected by the conformational change of brush polymers with high grafting density. The overlapping non-stretched linear chains occur due to the high density of polymer chains on surface, which increases the interaction among segments. Therefore, the grafting chain extends in a direction perpendicular to grafting surface, thus increasing the height of brush.

**Figure 3:**
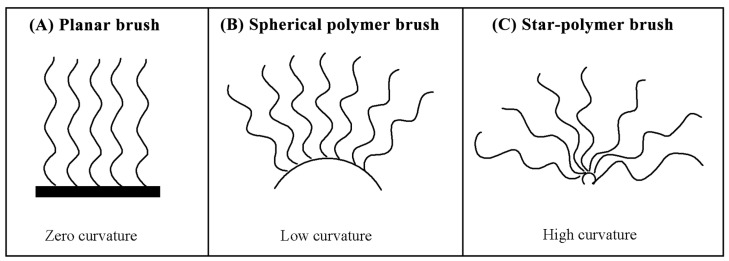
Curvature and structure of polymer brushes: (**A**) planar brush, (**B**) spherical polymer brush, (**C**) star-polymer brush.

Evaluation of conformation according to the curvature of grafted surface, polymer brush can be divided into three types: planar brush, spherical polymer brush and star-polymer brush (as shown in Figure 3). When the curvature radius of the grafted surface is far larger than the thickness of grafted brush layer, a planar brush is formed. A star-polymer brush is formed if the curvature radius of grafted surface is much less than the thickness of grafted brush layer. Spherical polymer brushes result with its thickness of grafted brush layer comparable to the curvature radius of grafted surface, which is a bridge to study the properties of polymer brush.

**Figure 4:**
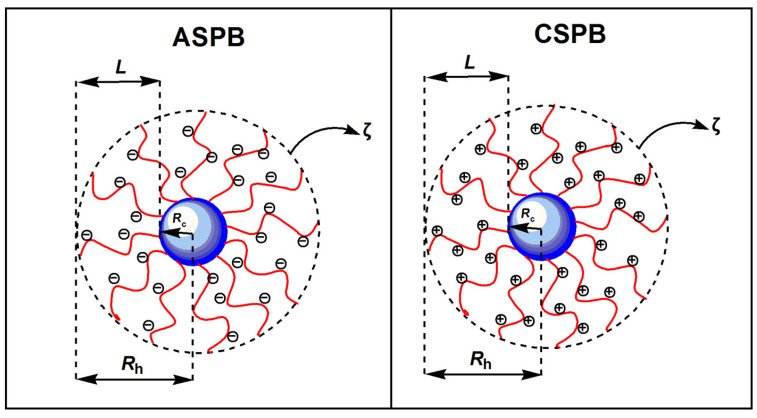
Structure diagrams of ASPB and CSPB. *L* denotes the thickness of brush layer, *R*_h_ the hydrodynamic radius, *R*_h_ the core radius, and *ζ* the zeta potential.

In order to extend polymer chains to form a thick polymer brush layer, the charged polyelectrolyte brush must attract more attention. In addition to the steric hindrance effect, charge repulsion and high osmotic pressure generated by counterions contribute to the stretching of polyelectrolyte chains. Therefore, the chain extension, brush layer thickness and conformation of polyelectrolyte brush are subject to pH, salt concentration, anti-ion valence and other external factors. According to the ionic types of grafted polyelectrolyte chains, spherical polyelectrolyte brushes (SPBs) could be divided into two classes: anionic spherical polyelectrolyte brushes (ASPB) and cationic spherical polyelectrolyte brushes (CSPB), as shown in Figure 4.

Before the 1990s, research on core-shell polyelectrolyte brushes was generally focused on polystyrene (PS) core with polymethyl methacrylate (PMMA) [85,86], polyacrylic acid (PAA) [87,88] shell. The application of PS, however, is limited due to its environmentally harmful chemicals. In view of this, novel green core materials such as silicon-based materials [89,90], carbon-based materials [91], graphene [92,93] and shell materials with various properties are emphasized for the research.

### 3.1. Synthesis Methods 

#### 3.1.1. Physisorption

**Figure 5:**
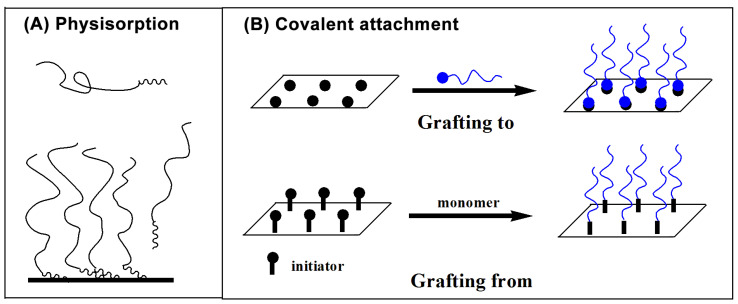
Schematic representation of (**A**) physisorption and (**B**) covalent attachment.

As shown in Figure 5A, physisorption refers to the self-assembly process of macromolecules with surface activity or polymers with end group functional groups [94]. Therefore, it is a reversible process. Both graft copolymers and block copolymers can be prepared by physisorption, whose essence is based on the selective solvation. It means that the behavior of insoluble and soluble segments of polymers varieties. The former settled down and affixed on the matrix, while the latter stretched, forming a polymer brush. In addition, when selective adsorption of graft copolymers happens on the substrate surface, polymer brushes can also be formed. Because of the weak hydrogen bond or van der Waals force in the process of physical adsorption, the desorption of adsorbed macromolecules takes places easily.

#### 3.1.2. Chemical Bonding 

Unlike physisorption, chemical bonding is an irreversible process, which means the polymer chains are attached by chemical bonds to substrate surface. As shown in Figure 5B, two technologies (“grafting to” and “grafting from”) can be used to describe this process.

“Grafting to” refers to the reaction between synthesized polymers with functional end-groups and substrate surface under appropriate reaction conditions. The polymer chains are then chemically grafted to the substrate surface though covalent bonds, thus forming a polymer brush [95]. The grafting density of brushes formed in this fashion is limited due to the steric hindrance among the preformed polymers.

“Grafting from” is performed on in situ polymerization initiated by the initiator under the light or heat [96,97]. Because of bearing initiator functionalities on substrate surfaces, the polymer brush prepared by this method has high grafting density. Depending on the initiators, the technique can be divided into several types: conventional free radical polymerization [98,99], active free radical polymerization [100,101], self-assembly microsphere system [102,103] or glow discharge treatment [104].

### 3.2. Characteristic Methods and Conformation

#### 3.2.1. Characteristic Methods

**Figure 6:**
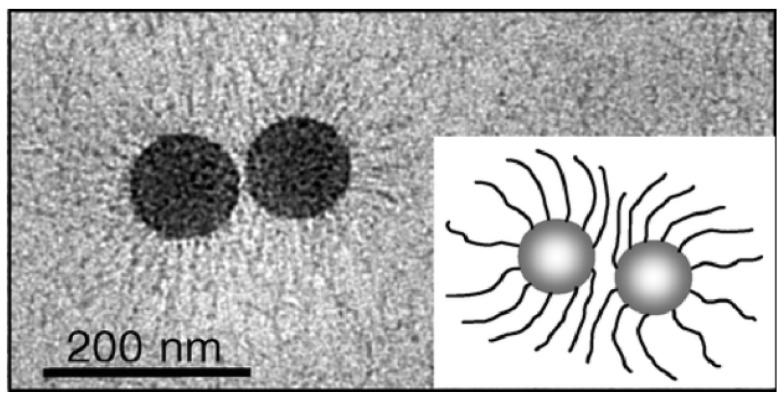
Cryo-TEM of spherical polyelectrolyte brushes. Particles consist of a core onto which a dense layer of poly(acrylic acid) chains has been grafted. Reproduced from ref. [105] with permission. Copyright 2005, American Chemical Society.

**Figure 7:**
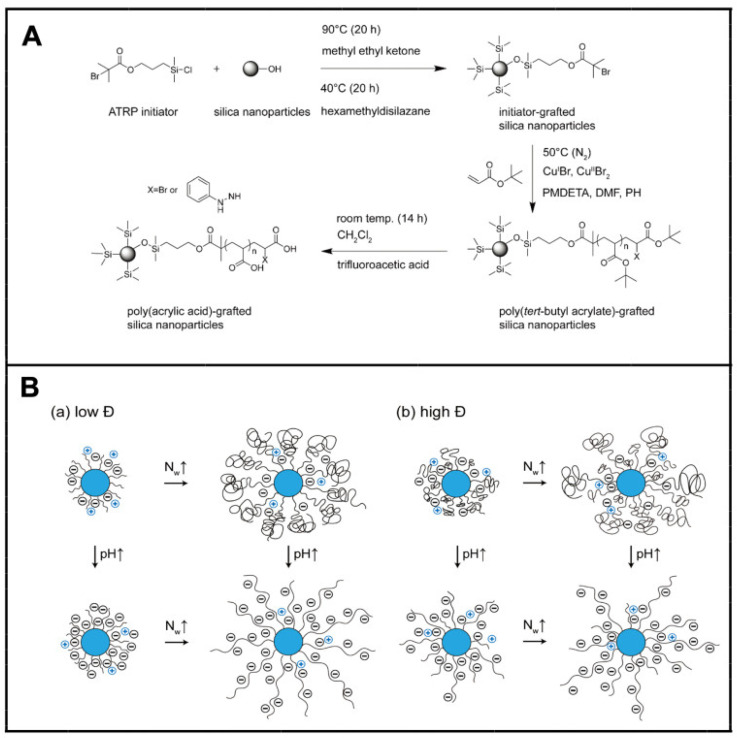
Synthesis of PAA brushes: (**A**) schematic representation of the conformation of annealed polyacid-grafted nanoparticles; (**B**) (a) low dispersity (Đ) and (**B**) (b) high dispersity (Đ) with variation of N_w_ and pH. “↑”denotes rising, “↓”denotes falling. Reproduced from ref. [112] with permission. Copyright 2021, The Royal Society of Chemistry.

As shown in Figure 6, the surface morphology of SPB can be characterized by low temperature transmission electron microscopy (cryo-TEM) [105,106], small angle neutron and X-ray scattering [107,108]. The particle size can be measured by atomic force microscopy (AFM) [109,110] and dynamic light scattering (DLS) [111]. The molecular weight and distribution of polymer brushes can be clipped from the grafted surface and then characterized by gel permeation chromatography (GPC) [112] (Figure 7), calculating the grafting density.

#### 3.2.2. Conformation 

In order to study the conformation of SPB, many theoretical models have been explored [113]. According to the complexity, it can be divided into three kinds: scale theory [114], numerical self-consistent field theory (NSCFT) [115], analytical self-consistent field theory (ASCFT) [116], molecular dynamics (MD) [117] and Brownian dynamics (BD) [118].

**Figure 8:**
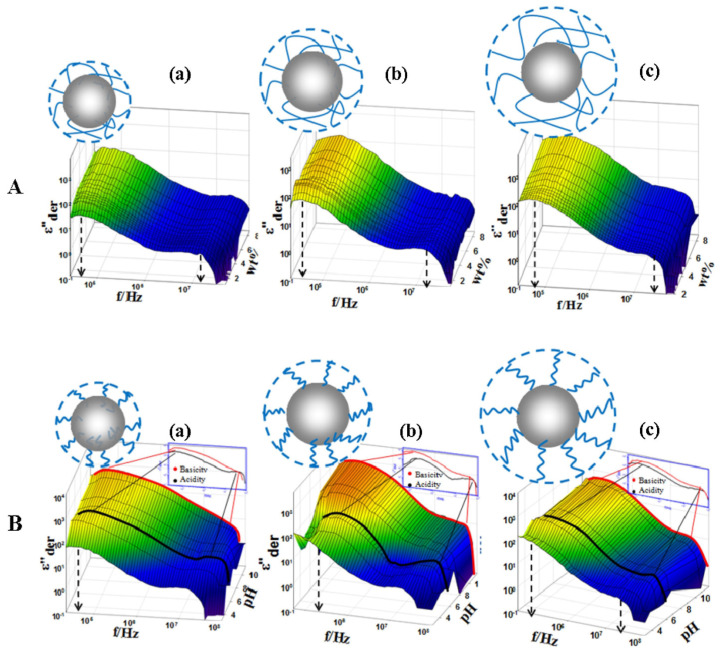
Three–dimensional representations of mass fraction dependence of derivative dielectric loss spectra (**A**) and pH dependence of the dielectric loss when the mass fraction is 2.32% (**B**) of PS–PAA, (a) (b) (c) are PS–PAA1, 2, 3, respectively. Reproduced from ref. [119] with permission. Copyright 2016, Elsevier B. V.

In addition, the influence of external factors (ionic strength, pH) on the conformational parameters of polyelectrolyte brush can also be monitored by modern physical testing methods [119] as shown in Figure 8.

## 4. Synthesis of Conducting Polymers Doped with SPB

### 4.1. Synthesis Methods of Conducting Polymers

#### 4.1.1. Chemical Oxidation Polymerization

**Figure 9:**
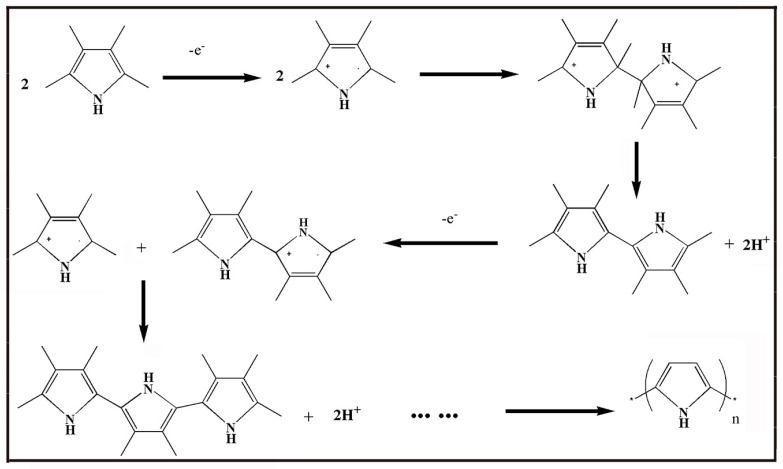
Schematic representation of synthesis process of PPy by chemical oxidation polymerization.

Conducting polymer powders are synthesized by chemical oxidation polymerization. Schematic of the synthesis process of PPy by chemical oxidation polymerization is shown in Figure 9. Firstly, an electrically neutral pyrrole loses an electron due to the action of an oxidant, thus becoming a cationic radical. Then, two cationic free radicals combine to dimeric pyrrole, which generates neutral dimeric pyrrole by disproportionation. By repeating the process, dipyrrole is oxidized to form trimer. Continue this cycle until a chain-like polymer with a degree of polymerization of n is generated. The monomers of conducting polymers are polymerized by incorporation of oxidants such as ferric chloride (FeCl_3_) [120,121], ammonium persulfate ((NH_4_) _2_S_2_O_8_, APS) [122,123,124] and hydrogen peroxide (H_2_O_2_) [125,126]. At present, conducting polymers are synthesized with different protonic acid dopants, namely hydrochloric acid (HCl) [127,128], sulfuric acid (H_2_SO_4_) [129,130,131] and perchloric acid (HClO_4_) [132,133]. However, these nonvolatile acids may remain on the surface of conducting polymers, thus affecting their performance.

Gao JW et al. [134] synthesized montmorillonite/polypyrrole (MMT/PPy) nanocomposites by in situ chemical oxidation polymerization (FeCl_3_ as oxidant). The content of PPy varied from 10 to 80 wt%, and the reaction temperature was about 0 °C. When the critical content (50 wt%) of polypyrrole was exceeded, the electrical conductivity of MMT/PPy nanocomposites was higher than that of PPy. For example, the electrical conductivity of PPy was 2.71 s/cm. When the content of PPy was 50 wt%, 60 wt% and 80 wt%, the electrical conductivity of MMT/PPy nanocomposites was 2.72 s/cm, 3.68 s/cm and 4.81 s/cm, respectively. Furthermore, the thermal stability of the nanocomposites was improved by the addition of MMT. Gu Z [135] prepared graphite/polypyrrole (GO/PPy) composites using hydrochloric acid as dopant. In comparison with GO, the electrical conductivity of GO/PPy composites increased by four orders of magnitude, and the thermal stability of GO/PPy composites improved. Wu TM et al. [136] synthesized composites with high electrical conductivity and good solubility by using APS as oxidant. Different concentrations of anionic polyelectrolyte sodium polystyrene sulfonate (PSS) were taken as dopants. Mass ratio of PSS/pyrole was 0.1, 0.15, 0.2, 0.25 and 0.3, respectively. The highest electrical conductivity was exhibited when the mass ratio of PSS/pyrrole was 0.25, which reached 151.3 ± 5.3 s/cm. Porramezan M and Eisazadeh H [137] synthesized PANI/Ag_2_O nanocomposites by using APS as oxidant and hydroxypropylcellulose as a space stabilizer. Results showed that Ag_2_O had a significant influence on the particle size and appearance of the nanocomposites, and the thermal stability of PANI was improved due to the existence of Ag_2_O. In 2009, Can M et al. [138] successfully synthesized polyaniline using periodic acid (H_5_IO_6_) as oxidant. After H_5_IO_6_ was reduced to IO_3_^−^, I_2_ was then formed. IO_3_^−^ and I_2_ could be separately used as an oxidant and dopant of aniline monomer. Therefore, the oxidation and doping processes were completed simultaneously. Its electrical conductivity was 100 S/cm.

In order to improve the water solubility of polyaniline, scholars have synthesized polyaniline by the aqueous–aqueous emulsion method. Rubinger C P L et al. [139] synthesized PPy/SiO_2_ nanocomposites by emulsion polymerization technique, using methyl cellulose as stabilizer and APS as oxidant. The conduction process can be described by three-dimensional variable-rang hopping (3D-VRH) model. Asim N et al. [140] used Hexadecy ltrimethyl ammonium bromide (CTAB) as the template to prepare PANI/V_2_O_5_ nanocomposite with core-shell structure by micro-emulsion polymerization. It was found that the thermal stability of the nanocomposites was better than that of polyaniline. Wang Y et al. [141] reported PANI/PS-PSS core-shell nanocomposites by in situ chemical oxidation polymerization of polystyrene polystyrene sulfonate (PS-PSS) copolymer on the surface of PANI. Results showed that the conductive composites can be formed when the mass fraction of PANI in the nanocomposite was approximately 2.78–12.5%, and the electrical conductivity was 1.7 S/cm.

#### 4.1.2. Electrochemical Oxidation Polymerization

Conducting polymer films can be directly deposited on the electrode by electrochemical oxidation polymerization [142,143,144]. Masa J et al. [145] synthesized metal-polypyrrole (M-PPy) nanocomposites by electrochemical oxidation polymerization. The synthesis process was carried out as follows: firstly, PPy was prepared on the glassy carbon electrode by electrochemical polymerization. Metal particles (M = Mn, Fe and Co) were then introduced alternately by electro-oxidation and reduction. It had been observed by electrochemical techniques of cyclic voltammetry (CV) and hydrodynamics rotating disk electrode (RDE) that M-PPy composites represented the ability of catalytic reduction of oxygen in acid medium. When the heat treatment temperature of the composite was 450 °C~850 °C, the activity of the composite increased significantly in the nitrogen atmosphere. Javier HF et al. [146] synthesized single-wall carbon nanotubes (SWCNTs)/polypyrrole composite films by electrochemical oxidation polymerization. The effects of SDBS and SWCNTs on the polymerization process were investigated, and results showed that the impedance coefficient of the electrode was decreased because of the existence of SDBS and SWCNTs, while the capacitance of the film was increased. The thickness, roughness and stiffness of the film also increased.

Since polyaniline film with electrical activity was first successful synthesized by Diaz AF [147] using electrochemical oxidation polymerization in 1980, many studies have been carried out on the electrochemical behavior of conducting polymers [148]. Electrochemical polymerization of aniline can be described in three steps. The first step is that aniline monomer loses electrons and turns into a positively charged free radical, which forms dimer with its resonance isomer. Then dimer loses electrons and turns into a free radical again under the electrochemical condition, forming a trimer with aniline free radical. In this way, the polymer chains grow continuously until PANI is formed and deposited on the anode.

Liu x et al. [149] synthesized PANI/SiO_2_ composite by electrochemical oxidation polymerization without electrolyte additive under the condition of pH = 1.0–2.1. Chowdhury A N et al. [150] synthesized conducting copolymer polyaniline/poly(toluidine)/silica (PANI/POT/SiO_2_) composite film on platinum electrode by electrochemical oxidation polymerization. Excellent electrical activity of synthesized film was showed by the addition of silica to the copolymers. Borole D et al. [151] investigated the effects of different organic acids (benzoic acid, cinnamic acid, oxalic acid, malonic acid, succinic acid and adipic acid) and inorganic acids (sulfuric acid, hydrochloric acid, nitric acid, phosphoric acid and perchloric acid) on the electrochemical synthesis of polyaniline, poly(toluidine) and their copolymerized membranes. It was found that above three conducting polymers can be formed in all inorganic electrolyte solutions and organic electrolyte solutions (oxalic acid). The current density of the three conducting polymers in the anode was affected by the anions in the solution, and the electrical conductivity was influenced by the type of electrolyte.

### 4.2. PANI Doped by SPB

Due to the complex structure of polyaniline, the benzene-quinone structural model of polyaniline proposed by MacDiarmid was not accepted until in 1987 [152]. Wang F. et al. [153] confirmed the existence of a quinone ring, and also proved that the ratio of benzene to quinone ring was 3:1 by analyzing IR and Raman spectra of polyaniline.

**Figure 10:**
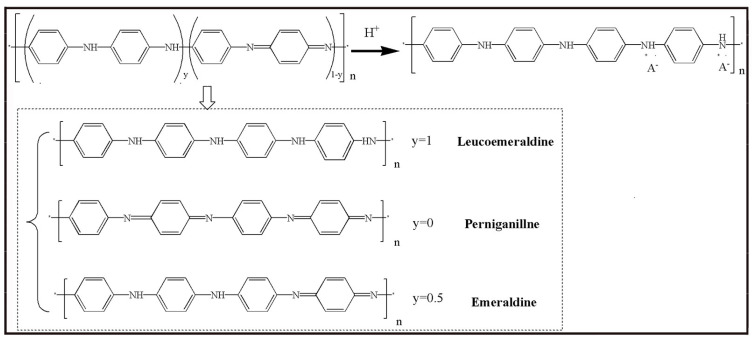
Scheme representation of redox status for polyaniline.

The intrinsic polyaniline is an insulator. The electrical conductivity of polyaniline can be increased by more than 10 orders of magnitude by proton acid or electrochemical doping. However, the doping mechanism of acid-doped polyaniline is different from that of other conducting polymers, whose doping process is always accompanied by the gain and loss of electrons. That is, no valence change for dopant occurs in the acid-doped polyaniline. In the process of doping, the nitrogen atom at the imine group is firstly protonated by H^+^, which led to the appearance of holes in the valence band of doped polyaniline (p-type doping). So a stable and delocalized poly(alexandrine imine) atomic group is formed. The positive charge of imine nitrogen atoms is dispersed along the molecular chain to the neighboring atoms through conjugation, thus increasing the stability of the system. Under the action of external electric field, the holes move on the polyaniline chains through the resonance of conjugated π-electron, showing the electrical conductivity. Figure 10 shows the structure of polyaniline changes with different redox status.

The benzene structure (Leucoemeraldine) (y = 1) and the quinone structure (Pernigraniline) (y = 0) are both insulators, which could not be changed into conductors through protonic acid doping. The benzene-quinone structure (0 < y < 1), which is called the intermediate oxidation state (Emeraldine), can be changed from insulators to conductors through protonic acid doping. Generally, when the ratio of quinone ring to benzene ring on polyaniline chain is 1:3 (y = 0.5), the best electrical conductivity of polyaniline is obtained.

**Figure 11:**
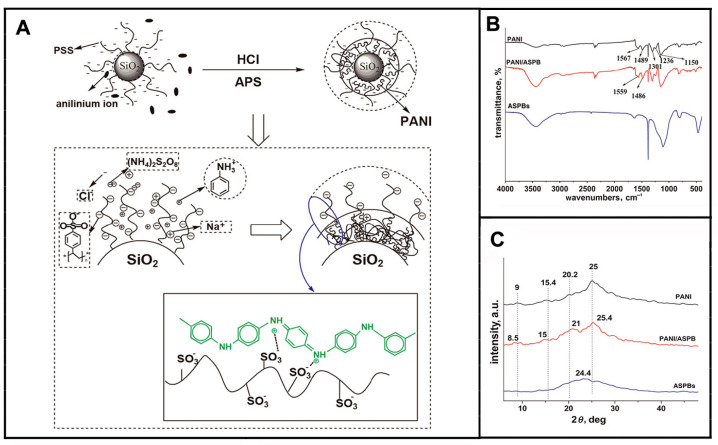
Scheme representation of doping mechanism of PANI (**A**); FTIR spectra of PANI, PANI/ASPB and ASPB (**B**); XRD patterns of PANI, PANI/ASPB and ASPB (**C**). Reproduced from ref. [155] with permission. Copyright 2014, The Institution of Engineering and Technology.

The properties of polyaniline are closely related to the preparation conditions. Studies have found that acidic system is more conducive to the occurrence of chemical behavior of polyaniline compared to alkaline and neutral systems. The suspensions of polyaniline-coated polystyrene microsphere were synthesized by Ke et al. [154]. Results from cyclic voltammetry test indicated that two oxidation peaks were observed, one of which (at 0.5 V) was attributed to the oxidation of polyaniline. Moreover, inconsistent chain length and cross-linking degree of polyaniline on the microspheres during synthesis resulted in low consequential recurrence by cyclic voltammetry test. It was essential to synthesize microspheres with active functional groups for exploring the doping mechanism of conducting polymers [155], as displayed in Figure 11.

Taking ASPB (PS core, PSS brush) as a template, Korovin et al. [156] prepared PANI-coated ASPB (ASPB-PANI) composites in 0.01 M hydrochloric acid using APS as oxidant. It was shown that with the concentration of NaCl ranging from approximately 10^−5^ M to 10^−1^ M, the zeta potential of synthesized composites lied between –40 mV and –80 mV, thus proving the excellent colloidal stability of SPB-PANI composites. Furthermore, the electrical conductivity of SPB-PANI composites improved because of the addition of ASPB.

### 4.3. PPy Doped by SPB

**Figure 12:**
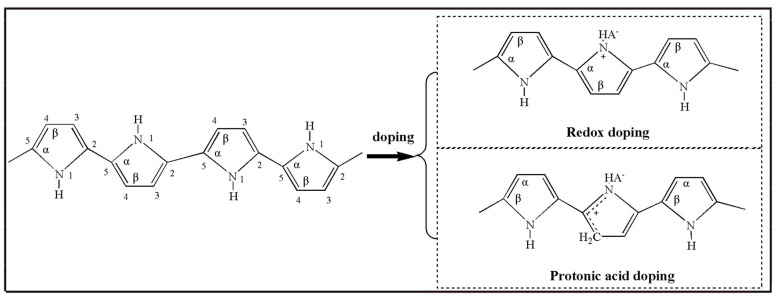
Scheme representation of PPy structure.

Polypyrrole plays an important role in conducting polymers because of its high electrical conductivity, easy synthesis and environmental friendliness. PPy is a semi-crystalline polymer by coupling with C 2 and C 5 of the pyrrole ring. Its structure is shown in Figure 12.

A conjugated structure consists of alternating C-C and C=C in PPy chains. Unlike σ electron in C=C which is fixed on carton atom by covalent bond, two π electrons in the conjugated double bonds can move on the entire molecular chains. That is to say, the energy band for the whole molecule is produced by the overlap of π electron cloud. The π electron moves in the molecular chains under external electric field, forming the electron conductivity of PPy. However, since a high degree of polymerization is required for activated carrier at room temperatures, poor electrical conductivity (10^−8^ S/cm) of pristine PPy is showed. Defects in its conjugated structure may be caused by doping, which enhances its electrical conductivity. The doping of PPy can be achieved by either protonic doping or redox doping (Figure 12).

In the process of protonic acid doping of polypyrrole, the protonation is firstly placed at β carton of pyrrole and then the positively charged protons are transferred to the polypyrrole chains. Meanwhile, the doping process is proceeding between negatively charged anion and polymer chains. A high degree of π-electron delocalization in the conjugated structure of PPy displays not only electrophilicity but also low electron dissociation energy. Under different reaction conditions, the polymer chain may be oxidized (lose electrons) or reduced (gain electrons), accompanied with dopant ions formed. Subsequently, the electrical neutrality of polymer is maintained by the electrostatic interaction of dopant ions and polymer chains.

**Figure 13:**
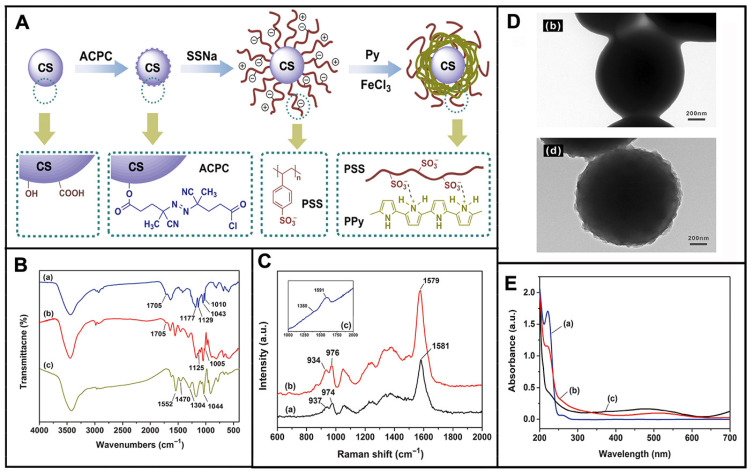
Scheme illustration of synthesis of PPy/ASPB composites (**A**); FTIR spectra (**B**) of (a) carbon spheres, (b) azo initiator-immobilized carbon spheres and (c) SPB; Raman spectra (**C**) of (a) PPy, (b) PPy/SPB composites and (c) SPB; TEM images (**D**) of (b) SPB and (d) PPy/SPB composites; UV-vis absorption spectra (**E**) of (a) SPB, (b) PPy/SPB composites and (c) PPy. Reproduced from ref. [157] with permission. Copyright: 2015 American Scientific Publishers.

Synthesis of PPy/ASPB composites was reported by Huang et al. [157] (Figure 13). A two-step process for the synthesis of ASPB was developed. Firstly, the carbon sphere’s core was synthetized by hydrothermal method. Polymerization was then initiated by azo initiator by the addition of sodium styrene sulfonate (SSS). It was found that ASPB played the role of the carrier of PPy.

### 4.4. PANI-PPy Doped by SPB

The physical and chemical properties of copolymers are obviously different from those of homopolymers and their blends [158]. Most conducting polymers or polymer composites lose electrical conductivity gradually due to their phase separation. Copolymers are relatively stable because different polymers are connected by covalent bond. It can be said that copolymer is a kind of multifunctional material which integrate the properties of different comonomers. Therefore, the study of aniline/pyrrole copolymer is not only of great theoretical significance but also practical application.

For the copolymerization of aniline and pyrrole, it can be described by two competing processes [159]. One is a primary interaction between comonomers, while the other is the secondary interaction between active aniline, active pyrrole and active aniline-pyrrole. In comparison with extensive reports of polypyrrole and polyaniline, research on poly(aniline-co-pyrrole) are far from being adequate. Up to now, most of the previous research has focused on the topic of synthesized copolymers with nanostructures using various templates [160] including poly(aniline-co-pyrrole) nanospheres synthesized by chemical copolymerization [161] and poly(aniline-co-pyrrole) nanocomposites coated on carbon fibers surfaces though one-step electrochemical method [162]. As reported by Huang [163] et al., the room temperature conductivity of poly(aniline-co-pyrrole)/ASPB composites (8.3 S/cm), which were prepared by chemical oxidation polymerization method, was higher than that of un-doped poly(aniline-co-pyrrole) (2.1 S/cm).

## 5. Research Methods

According to the theory of Su, Schridffer and Heeger (SSH) [164] in 1979, the carriers of conducting polymers are mainly composed of polaron, bipolaron and soliton. In order to study the structure and properties of conducting polymers, as well as their conducting and doping mechanism, various research methods involving spectroscopy, morphology, cyclic voltammetry, conductivity and transient current are widely used.

### 5.1. Spectra Analysis

#### 5.1.1. FTIR

Carrasco P M and Grande H J et al. [165] studied the relationship between the conjugate length and the electrical conductivity of conductive polymers using FTIR. A graphic illustration of the ratio of the integrated absorption areas of the 1445 cm^−1^ and 1535 cm^−1^ (A_1445_/A_1535_) as the abscissa axis and log (Conductivity) as the ordinate axis suggested that the larger the slope, the smaller the conjugate length based on the theory proposed by Baughman R and Shacklette L [166]. Nicho M E and Hu H [167] investigated the coating of PPy composites using infrared spectroscopy. It was proved that, on one hand, the interaction between the functional groups of polyvinyl alcohol and the iron atom of FeCl_3_. On the other hand, it was suggested that the conductivity of PPy was related to chloride ion. Thus, the chloride ions were associated with the conductive part and insulation part in conductive coating. The dispersion of PPy in the polymers is owing to the interaction among molecules. David W and Hatchett et al. [168] studied the acid-doped PANI by infrared spectroscopy. It was found that the strong acid and weak acid had a fundamental difference in doping principle. The oxidation state of polymers was stabilized by the sustained-release system caused by weak acid. On the contrary, the sustained-release system was not provided by strong acid, resulting in the redox state changing with washing process.

#### 5.1.2. UV-Vis

Reported by Shen YQ and Wan MX [169], the soluble PPy doped with β-naphthalenesulfonic acid was analyzed by UV-Vis spectroscopy. The difference of doping degree was proven by the shift of the peaks of polaron and bipolaron in the UV-Vis spectroscopy. Similarly, in the PPy film prepared by electrochemical oxidation polymerization, the movement of peak depended on potential, demonstrating the interaction among polymer chains. Malinauskas A and Holze R [170] studied the degradation of polyaniline using UV-Vis spectroscopy and discussed the variation of spectrum at high potential. The degradation followed the first order kinetics at the electrode potential from +0.85 to +1.20 V, and the rate constant ranged from 8.40 × 10^−6^ to 2.93 × 10^−3^ s^−1^. Pruneanu S et al. [171] studied the structure and properties of PANI films formed by electrochemical oxidation polymerization using UV-Vis spectroscopy. It was found that the absorbance of films formed in N-methylpyrrolidone (NMP) solution increased with the increase of the deposition times of NMP. UV-Vis spectroscopy results demonstrated that the degradation of polymers was prevented by thick films.

#### 5.1.3. Raman

Claudio H and Silva B et al. [172] characterized the polymerization products of Ani-APS in aqueous solution at 413.1 nm and 1064 nm in Raman spectra at different pH values. It was found that the polymer product was PANI-ES at pH = 4.9, and the characteristic absorption peak appeared at 1064 nm in Raman spectroscopy. At pH = 13.2, the characteristic peaks at 1064 nm and 413.1 nm indicated that the main product was adduct of 1, 4-Michael aniline monomer and 1, 4-benzoquinone-monoimine. Lee S et al. [173] studied the electrical and optical properties of the polyaniline nanowires between the metal and the insulator (M–I transition). When the pH value of the solution was 2, the electrical conductivity of the polyaniline nanowires was reduced to 0.95 S/cm. This de-doping behavior can be characterized by Raman spectroscopy.

#### 5.1.4. XPS

**Figure 14:**
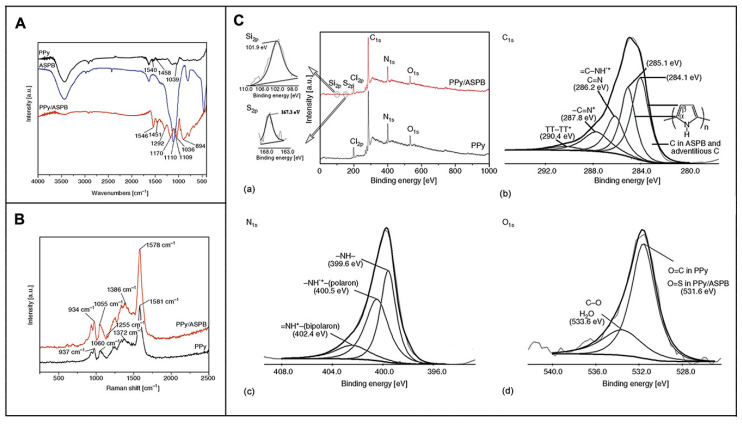
FTIR spectra of (**A**) PPy, ASPB, PPy/ASPB; Raman spectra of (**B**) PPy/ASPB and PPy; (**C**) XPS spectra of (a) wide region spectroscopy, (b) C_1s_, (c) N_1s_, (d) O_1s_ of PPy/ASPB nanocomposite. π−π* represents the transition from ground state (π) to excited state (π*). Reproduced from ref. [175] with permission. Copyright: 2012 BME–PT.

X-ray photoelectron spectroscopy, which can give approximately 5–10 nm surface atomic composition and chemical bond characteristics, can be used to analyze the doping process including doping degree N^+^/N, crosslinking degree, energy distribution and atomic valence. Therefore, XPS is the most commonly used method for studying the structure and doping degree of conducting polymers. Lin et al. [174] studied the effects of PANI-HCl, PANI-SDBS-HCl and PANI-DBSA on different PANI doping systems by XPS. Results showed that the PANI in PANI-HCl system was prone to de-doping in the process of post-treatment. In PANI-SDBS-HCl system, DBS^−^ was combined with positively charged provided by PANI chains due to electrostatic adsorption, which acted as a dopant and simultaneously induced the dissolution of PANI. Moreover, the spectra of N_1s_ and S_2p_ of PANI-SDBS-HCl was similar to that of PANI-DBSA, but the N^+^/N and S^−^/N of SDBS-HCl were 0.46 and 1.14, respectively, indicating optimum doping level. As we reported earlier [175], PPy/ASPB nanocomposite was synthesized by in situ chemical oxidative polymerization. Information about its structure was characterized by FTIR, Raman spectra and XPS measurements (Figure 14).

### 5.2. Morphological Analysis

**Figure 15:**
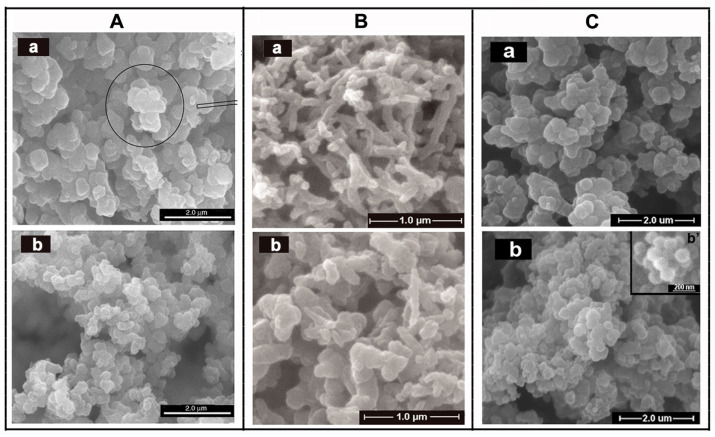
SEM images of (**A**) PPy (a), PPy/ASPB nanocomposite (b), reproduced from ref. [176] with permission. Copyright: 2012 BME-PT; (**B**) PANI (a), PANI/ASPB nanocomposite (b), reproduced from ref. [155] with permission. Copyright: The Institution of Engineering and Technology; (**C**) PPy–PANI (a), (PPy–PANI)/ASPB composites (b), reproduced from ref. [183] with permission. Copyright: 2012 BME–PT.

In general, the characterization methods of the morphology of conducting polymers are mainly TEM, SEM and FE-SEM (Figure 15). For example, the microstructure of un-doped PPy usually appears as a cauliflower-like or tumor-like structure [176]. It is mainly due to the similar polymerization capacity of α-carbon and β-carbon of pyrrole monomers, leading to the performance of three-dimensional polymer growth. The addition of dopants can provide the spatial factors for the orderly growth of pyrrole, resulting in polypyrrole with a special microstructure, such as dendritic [177], fibrous [178] and tube [179]. Wang Y et al. [180] prepared PPy with helical and cyclic microstructures using APS as the oxidant and cetyltrimethylammonium bromide as the cationic surfactant. The appearance of PANI is generally presented rod-like or fibrous, mainly due to inherent characteristics of fibrous chain growth in aniline polymerization reaction [181]. Using this characteristic, Yang S M et al. [182] prepared PANI fibers with different particle sizes in the presence of anodic aluminum oxide template in different voids using chemical oxidation polymerization and electrochemical oxidation polymerization. Figure 15 displays the morphologies of PPy/ASPB, PANI/ASPB and (PPy-PANI)/ASPB composites [183] by SEM.

In addition, the crystallinity of conducting polymers is significant, which is directed by X-ray diffraction (XRD) [184,185,186]. Pruneanu S et al. [187] studied the PPy films doped by perchlorate (ClO_4_^−^) and p-benzenesulfonic acid (TsO^−^) using electrochemical method by XRD. It was found that anion type had an important effect on the oxidation behavior and structure of polymers. The dynamic parameters (AC charge density, cathodic transfer coefficient and anode transfer coefficient) were obtained using the Fiat formula. It was also found that the PPy film doped with ClO_4_^−^ had high anisotropy. In contrast, the PPy film doped with TsO^−^ exhibited isotropic. Pouget J P et al. [188] studied the two structures of polyaniline using XRD techniques, which laid a good foundation for research on the doping of aniline and the structure of polymers in the future.

### 5.3. Performance Tests

In general, thermal stability and solubility are commonly used to investigate the overall performance of conducting polymers. The electrical conductivity of conducting polymers depends on synthetic method, synthesis conditions, chain structure and the type and doping degree of dopants. The electrical conductivity at room temperature varies generally from 10^−9^ to 10^5^ S/cm. The four-probe method is the most direct and effective method for testing the electrical conductivity of conducting polymers.

**Figure 16:**
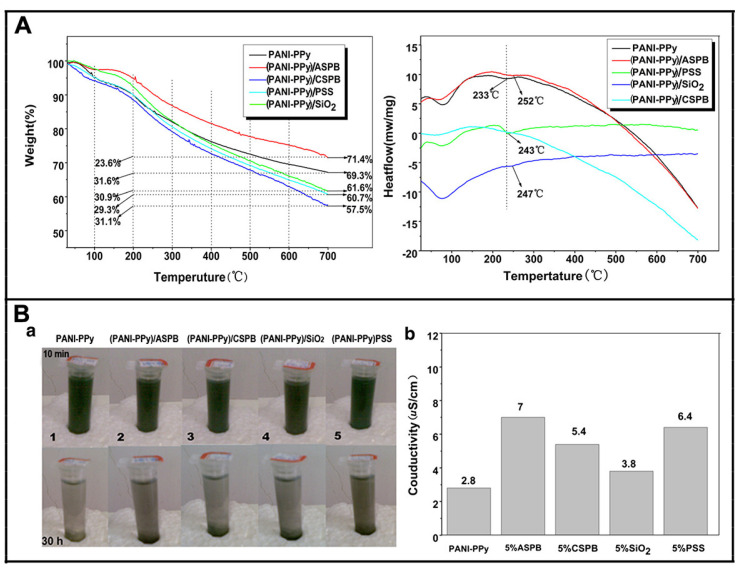
(**A**) Effect of polymerization temperature and molecular weight of grafted polyelectrolyte brushes on electrical conductivity; (**B**) qualitative (a) and quantitative (b) analysis of the conductivity of saturated solution of PANI–PPy nanocomposite with different dopants (T = 25 °C, Ph = 6). Reproduced from ref. [183] with permission. Copyright: 2012 BME–PT.

One of the limited factors of conducting polymers in practical application is its poor thermal stability, especially in the presence of moisture and oxygen. Thiéblemont J et al. [189] studied the oxidation of PPy powders in the air. It was found that when the temperature was higher than 230 °C, the oxidation process was obvious, leading to the decomposition of polymer. When the temperature was less than 230 °C, the oxidation process was slow and the kinetic process of oxidation process was investigated. The activation energy was about 110 kJ mol^−1^. The electrical conductivity of the conducting polymers decreased as oxidation degree increased. Therefore, in order to improve the thermal stability of conducting polymers for application needs, surfactants [190,191], metal complexes [192] and other substances were added [193,194]. We also studied the thermal stability and the conductivity of the saturated solution of ethanol used as reference (Figure 16). It was found that by doping ASPB, the thermal stability and solubility of PANI-PPy composite were enhanced.

In addition, electrochemical research methods, including cyclic voltammetry (CV) [195,196], electrochemical impedance spectroscopy (EIS) [197,198] and transient current (TC) [199,200] are commonly used.

## 6. Theory of Conducting Polymers Doped by SPB

### 6.1. Doping and Conducting Mechanism of Conducting Polymers

From the view of conducting mechanisms, conjugate double bond of organic polymers offers favorable factors of freedom for the movement for electrons. The presence of defects in the conjugated structure of conducting polymers helps to improve the conductivity. It is the process of removing (or adding) electrons from the polymer chains which is called doping. Taking redox doping and proton acid doping for example, the general mechanism is explained as follows:

Due to high delocalization of π electrons in the conjugated structure of conducting polymers, the conjugated polymer chains may be oxidized or reduced. Meanwhile, dopant ions are formed by the reduction or oxidation of dopant. The interaction between dopant ions and polymer chains is present to maintain the electrical neutrality of the polymer system. This is redox doping.

During the doping process of proton acid, no electron migrations happen between the polymer chains and dopants. Instead, the protons of dopants are attached to the carbon atoms of polymer chains. As a conjugated polymer chain expands, the charge distribution on polymer chain changes. However, not every kind of protonic acid follows this doping mechanism, especially for strong oxidation protonic acids, whose doping mechanism needs further study.

In addition, from the physics point of view, changes of the occupation of electrons happen in the molecular orbital of conducting polymers, which changes the π-electron energy levels and reduces the energy difference. This makes the resistance of carrier migration decrease, thus achieving high electrical conductivity. In contrast, wide energy gap lies on conducting polymers without doping. Basically, there is no electron on the anti-bond orbital at room temperature, resulting in low electrical conductivity.

### 6.2. Three-Dimensional Variable Range Hopping (3D VRH) Theoretical Models

Based on studying the macroscopic properties of materials, Granular Metal Islands Model is the most common theory that explains the conducting mechanisms of polyaniline [201]. According to the theory of Granular Metal Islands Model, conductive phase and an insulating phase polyaniline are formed with an increase in the degree of protonation. The conduction behavior of the conductive phase is realized through the limitation of tunnelling effect. The insulating phase is composed of materials defects, chain segments and linked and transitional doping regions. This model fully considers the anisotropy and internal heterogeneity of conducting polymers. It is considered that the entire conductive system consists of metal regions and surrounding insulating regions. The macroscopic conductivity is relevant to the interchain conductivity. Granular Metal Islands Model is proposed based on the relationship between electrical conductivity and temperature of doped PANI, as shown in Equation (1):

(1) $$\sigma =\sigma_{0}\exp[-{\left(\frac{T_{0}}{T}\right)}^{\frac{1}{r+1}}]$$

The parameter *σ*_0_ and *T*_0_ are depended on the molecular vibration frequency, localization length, average transition length and state density of materials. *r* (*r* = 1, 2, 3) stands for the dimension of electronic variable transition. Logarithmic Equation (2) is another transformation of Equation (1), which means a linear relationship between ln*σ* and T^−1/2^ and T^−1/4^.

(2) $$\ln\sigma =\ln\sigma_{0}-{\left(\frac{T_{0}}{T}\right)}^{\frac{1}{r+1}}$$

**Figure 17:**
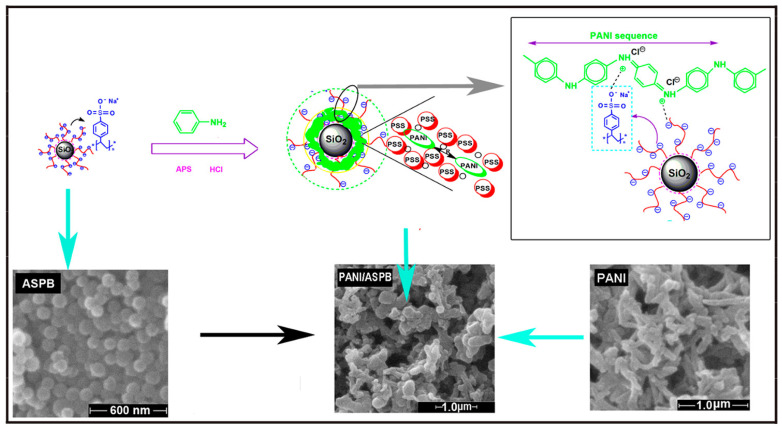
Schematic representations of reaction process and doping mechanism of ASPB. Reproduced from ref. [202] with permission. Copyright: 2015 MDPI.

As reported by literature, the conducting mechanism of PANI/ASPB can be explained by the three-dimensional variable range-hopping model (3D VRH) [202]. As displayed in Figure 17, SEM images show that PANI is fibrous, while PANI/ASPB displays similar circular particles whose diameter is between 100 nm and 500 nm. Assuming that each particle is crimped by a bundle of one-dimensional PANI chains, carrier mobility is closely correlated to one-dimensional molecular chains, which is three-dimensional variable range-hopping.

**Figure 18:**
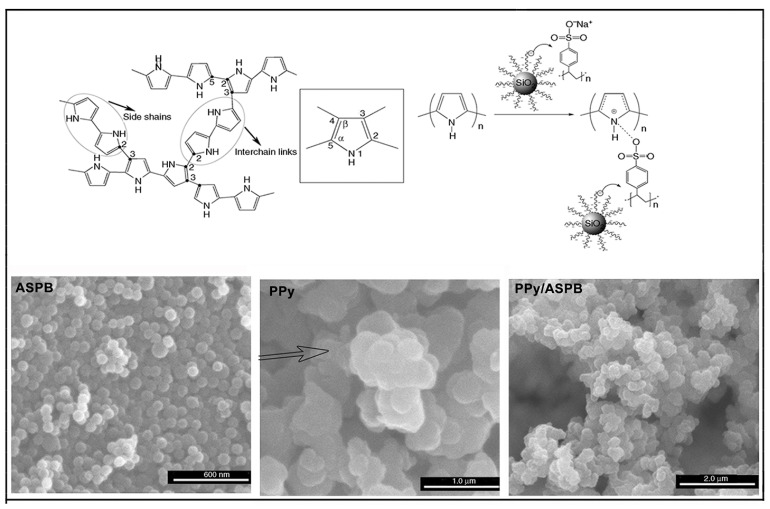
Schematic representations of formation mechanism of PPy/ASPB nanocomposite. Reproduced from ref. [175] with permission. Copyright: 2012 BME–PT.

Similarly, the morphologies characterized from the SEM images show that PPy/ASPB typically appears as a “cauliflower-like” or “tumor-like” structure (see Figure 18). The three-dimensional growth of PPy is achieved by the 2, 3-coupling polymerization, and the space factors for Py orderly growth is provided by the addition of ASPB with uniform spherical structure, which is demonstrated by the 3D VRH.

### 6.3. Template Theory

**Figure 19:**
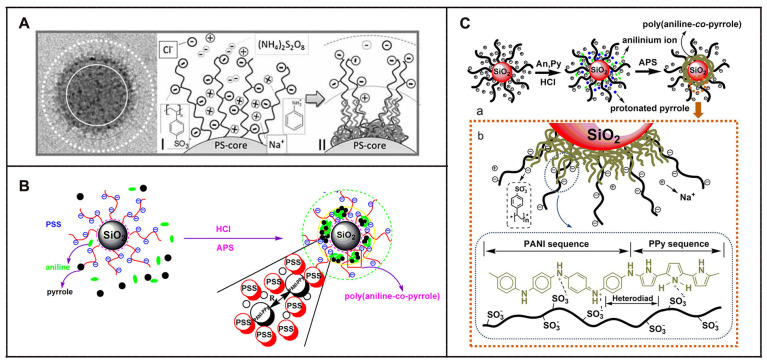
(**A**) Template of SPBs, reproduced from ref. [156] with permission. Copyright: 2011 John Wiley and Sons. (**B**) Doping mechanism of ASPB, Reproduced from ref. [183] with permission. Copyright: 2012 BME–PT; (**C**) Schematic representations of the synthesis of poly(aniline-co-pyrrole)/ASPB nanocomposites (a), the interaction between poly(aniline-co-pyrrole) and ASPB (b), reproduced from ref. [203] with permission. Copyright: 2014 John Wiley and Sons.

As an effective method to prepare nanomaterials, template method is characterized by the fact that chemical reactions are performed in an effectively controlled region, regardless of whether they are in liquid or gas phase. Therefore, the conformation and properties of nanomaterials can be precisely controlled using template as the carrier. Many investigators [156,175,203,204] explored the polymerization of aniline which is performed within the interfacial volume of SPBs. Because aniline monomers were confined efficiently within the finite volume of polyelectrolyte brushes, the optimal conditions for matrix polymerization were provided by SPBs (Figure 19).

## 7. Conclusions and Outlook

In this review, several conducting polymers doped by ASPB are presented. The doping of ASPB has greatly improved the conductive properties, thermal stability and solubility properties of conducting polymers. However, in view of unique chemical structure of ASPB, it still has significant scope to the development of conducting polymers doped with ASPB in future research.

On one hand, in the synthesis of ASPB, ASPB with controllable molecular structure was prepared by controlling the reaction condition parameters. Along with the molecular weight of grafted polymer chain, further in-depth research is needed to investigate the structure–activity relationship between the molecular structure of ASPB, such as graft density, charge density and the conductive properties of conducting polymers. Moreover, no further research has been conducted on the effect of the interaction between ASPB and conducting polymers on the performances of composites. On the other hand, except for PANI and PPy, more conducting polymers require further study. As for poly(aniline-co-pyrrole), present research only involves the equimolar ratio of two monomers. With different ratios of the copolymers studied, conducting polymers doped with ASPB will be further understood.

**Figure 20:**
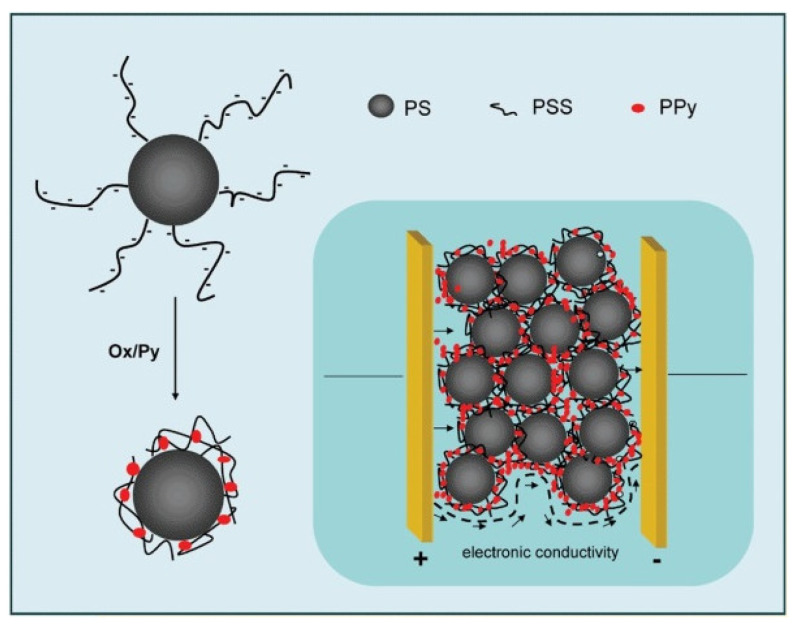
Formation of network of conducting domains within insulating matrix by loading the SPBs with PPy, reproduced from ref. [205] with permission. Copyright: 2009 John Wiley and Sons.

Wang et al. [205] developed the preparation of PPy/ASPB composites in organic electronic devices (Figure 20). The ASPB were used as the carrier of PPy, resulting in conductive ink good film-forming performance. Compared with PEDOT/PSS, PPy/ASPB composites have low leakage current, which opened up perspectives for the application in electrochemistry.

It is worth noting that biomacromolecules such as nanocellulose [206,207] polydopamine (PDA) [208,209] have been taken as novel dopants for conducting polymers, and the composites can be prepared by electrochemical polymerization.

In conclusion, although conducting polymers doped with SPBs have achieved significant progress, there are still many challenges that need to be addressed for the application in the future. In current research, high electrical conductivity poses a significant challenge for conducting polymers used for electronic and energy applications. Developing novel SPBs may represent a strategy to address this issue. Additionally, the preparation of conducting polymers doped with SPBs is limited to laboratory settings. Commercial production is still not viable.

## Data Availability

Data are contained within the article.
